# The effect of multiple genetic variants in predicting the risk of type 2 diabetes

**DOI:** 10.1186/1753-6561-3-s7-s49

**Published:** 2009-12-15

**Authors:** Qing Lu, Yeunjoo Song, Xuefeng Wang, Sungho Won, Yuehua Cui, Robert C Elston

**Affiliations:** 1 Department of Epidemiology, Michigan State University, B601 West Fee Hall, East Lansing, Michigan 48824 USA; 2 Department of Epidemiology and Biostatistics, Case Western Reserve University, 2103 Cornell Road, Cleveland, Ohio 44106 USA; 3 Department of Statistics and Probability, Michigan State University, A413 Wells Hall, East Lansing, Michigan 48824 USA

## Abstract

While recently performed genome-wide association studies have advanced the identification of genetic variants predisposing to type 2 diabetes (T2D), the potential application of these novel findings for disease prediction and prevention has not been well studied. Diabetes prediction and prevention have become urgent issues owing to the rapidly increasing prevalence of diabetes and its associated mortality, morbidity, and health care cost. New prediction approaches using genetic markers could facilitate early identification of high risk sub-groups of the population so that appropriate prevention methods could be effectively applied to delay, or even prevent, disease onset.

This paper assessed 18 recently identified T2D loci for their potential role in diabetes prediction. We built a new predictive genetic test for T2D using the Framingham Heart Study dataset. Using logistic regression and 15 additional loci, the new test was slightly improved over the existing test using just three loci. A formal comparison between the two tests suggests no significant improvement. We further formed a predictive genetic test for identifying early onset T2D and found higher classification accuracy for this test, not only indicating that these 18 loci have great potential for predicting early onset T2D, but also suggesting that they may play important roles in causing early-onset T2D.

To further improve the test's accuracy, we applied a newly developed nonparametric method capable of capturing high order interactions to the data, but it did not outperform a logistic regression that only considers single-locus effects. This could be explained by the absence of gene-gene interactions among the 18 loci.

## Background

Diabetes mellitus has become one of the major global health issues in the twenty-first century. In the past few decades, the incidence of diabetes has risen rapidly. Owing to its dramatically increased prevalence and associated high morbidity, mortality, and health care cost, early detection and prevention of diabetes have become critical. The most common form of diabetes is type 2 diabetes (T2D), which accounts for over 90% of all cases of diabetes. Although no established cure has been found for T2D, there is evidence that the progress of T2D can be largely prevented through exercise and diet control. In a large-scale study conducted in the United States, lifestyle intervention (i.e., diet control and exercise) has been shown to reduce the risk of T2D by 58% among high-risk individuals over 3 years [1].

Detecting T2D at its earliest stage is the key to more effectively applying lifestyle intervention to reduce morbidity and mortality. Recent research has focused on the genetic prediction of T2D (i.e., using a predictive genetic test). A predictive genetic test, which uses genetic markers (e.g., single-nucleotide polymorphisms, or SNPs) to predict an individual's future risk of disease, can be conducted early in life (e.g., at birth) and thus form one of the most appealing early disease prediction methods. Based on three widely replicated genetic variants, rs5219 (Glu23Lys) of *KCNJ11*, rs1801282 (Pro12Ala) of *PPARG*, and rs7903146 of *TCF7L2*, Weedon et al. [2] formed one of the earliest predictive genetic tests for T2D. Although the classification accuracy of the test, as measured by the area under the receiver operating characteristic (ROC) curve (AUC), is relatively low (0.58), this is an important step toward a successful predictive genetic test for T2D. With the current extensive genetic research in T2D, especially with the completion of multiple genome-wide association studies (GWAS), the number of confirmed genetic loci for T2D has been significantly increased. Recently, in the Tayside population of Scotland, Lango et al. [3] combined 18 T2D loci to form the most up-to-date predictive genetic test and found that the performance of the test was slightly improved (AUC = 0.6). A similar 18-locus test (AUC = 0.6) was also formed by another study conducted in a Rotterdam suburb population [4]. This latter study suggested a significant improvement of the new test (95% CI of the AUC is 0.57-0.63) over that of the three-locus test (95% CI of the AUC is 0.5-0.55). It should be noted that the estimated classification accuracy of the three-locus test obtained in the Rotterdam suburb population is lower than that in the other studies [2,3]. If we use the result from Weedon et al. (i.e., AUC = 0.58) instead of the one from the Rotterdam suburb population for the three-locus test, then the improvement is not significant.

We selected 18 T2D SNPs from the Framingham Heart Study (FHS), among which 11 are the same and 7 have alleles in high linkage disequilibrium with the 7 other SNPs used in Lango et al.'s study. We combined these 18 SNPs to form a new predictive genetic test, and compared it with the previous three-marker test [2] through hypothesis testing to determine whether it is a significant improvement in the FHS data. Two methods were used to form the test: one was logistic regression, only considering marginal single locus effects, and the other was our newly developed nonparametric method [5], which has the ability to capture high-order interactions. Based on these 18 T2D SNPs, we also investigated genetic tests for predicting early- and late-onset T2D, because previous evidence has suggested an important genetic influence on early-onset T2D, but a high-phenocopy rate for late-onset T2D [6].

## Methods

### Logistic regression vs. optimal robust ROC curve method

We used both logistic regression (implemented as a generalized linear model with a logit link) and a newly developed nonparametric method [5] to build the T2D predictive genetic test. Logistic regression is the most commonly used method for predictive genetic tests and has been used in the previous two studies [3,4] to evaluate the classification accuracy of the 18-locus test. In our logistic regression analysis, we adopted a model similar to that used by Lango et al. [3], which considers only single-locus effects:

where *β*_*i *_is the regression coefficient for the *i*^th ^genetic variable and *μ *is the probability of an individual developing T2D. Since, as indicated by Lango et al. [3], all the variants appear to follow an additive model, here we coded each genetic variant as additive instead of using two indicator variables for the three genotypes. Based on the fitted model, we formed the ROC curve and estimated the AUC. The logistic regression analysis was conducted using the *glm *function in the statistical package R.

The above logistic regression does not take into account any interaction effect, and may result in a low performance if there is a strong interaction effect. Alternatively, we can fit a logistic regression with interaction effects. However, for 18 loci a logistic regression model with all two-way interactions requires 171 parameters and fitting a model with such a large number of parameters will lead to biased estimates and an inflated type 1 error rate [7]. Unlike logistic regression, nonparametric methods have the feature of avoiding the issue of an increasing number of parameters, and thus have an advantage for identifying high order interactions. We recently developed a nonparametric method of combining multiple genetic variants for disease prediction [5]. The new method, named the optimal robust ROC curve method, was developed based on the optimality theory of the likelihood ratio. Such a theory simply shows that the ROC curve based on the likelihood ratio has maximum performance at each cutoff point and that the AUC so obtained is the highest among those of all approaches [8,9]. We incorporated a backward-clustering algorithm into the method. The backward-clustering algorithm automatically approaches the marker's disease model (i.e., mode of inheritance including interactions), eliminating the "noise" markers, and thus makes the approach robust to a variety of underlying disease models. The new method is implemented using the R program we developed.

Hypothesis testing

In Lango et al.'s study [3], the test based on 18 loci was found to have a slightly higher AUC than the previous three-marker test. Using the FHS dataset, we investigated whether the new test built on 18 loci had a significant improvement. To compare the accuracy of the new test (*A*) with that of the previous three-marker test (A_0_), we set up a hypothesis of interest (H_0 _: A = A_0_) and constructed an appropriate test statistic to test the hypothesis:

Under the null hypothesis, this test statistic follows a standard normal distribution in a large sample. The variance of the estimated AUC, , can be calculated as

where *n*_*D *_and  are respectively the numbers of the disease and non-disease subjects, var_*D *_and  and are simple functions of the sensitivity and specificity from the ROC curve [9].

## Results

We included 252 unrelated diabetes cases from the 252 diabetes families (i.e., the maximum number of unrelated cases), and 979 unrelated controls, one from each of the remaining families. Diabetes cases with age of diabetes onset less than 40 years were excluded from the study to eliminate patients with possible type 1 diabetes. We took the 18 loci used in Lango et al's study; we extracted 11 SNPs from the 500 k GWAS dataset and the 50 k candidate-gene dataset, and replaced the remaining seven SNPs that were absent from either dataset with existing SNPs that were in strong linkage disequilibrium with the SNPs used in Lango et al.'s study (Table 1).

**Table 1:** Summary of the seven original SNPs from Lango et al. [3] for which seven other SNPs were substituted in our study

Gene/region	Original SNP		Substituted SNP		LD	
			
	RS number	MAF	RS number	MAF	*D*'	***R***^2^
*TCF7L2*	rs7903146	0.30	rs4132670	0.30	1.00	0.92
*KCNJ11*	rs5219	0.36	Rs5215	0.33	1.00	0.99
*SLC30A8*	rs13266634	0.30	rs11558471	0.39	1.00	0.95
*CDC123*	rs12779790	0.20	rs7069060	0.23	1.00	0.63
*WFS1*	rs10010131	0.40	rs10012946	0.42	1.00	1.00
*TCF2*	rs757210	0.37	rs2107131	0.36	0.57	0.11
*HHEX-IDE*	rs1111875	0.38	rs5015480	0.44	1.00	1.00

**Figure 1:**
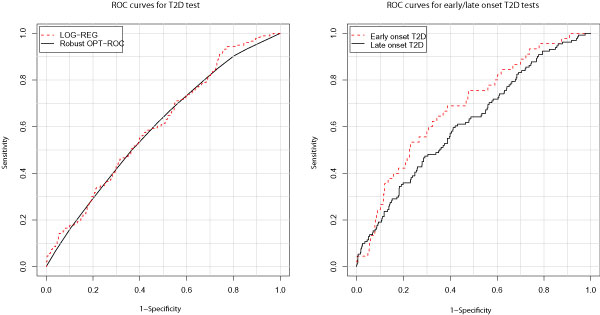
**ROC curves for T2D tests, and for early and late onset T2D tests**. The dashed and solid lines on the left panel correspond to the ROC curves of T2D tests built by the additive logistic regression model (AUC = 0.606) and the robust optimal ROC curve method (AUC = 0.596). In the right panel, dashed lines correspond to the ROC curves for tests to detect early-onset T2D (AUC = 0.683); solid lines correspond to the ROC curves for tests to detect late-onset T2D (AUC = 0.616).

Based on these 18 loci, we constructed predictive genetic tests using both the additive logistic regression model and the robust optimal ROC curve approach. The performance of the predictive genetic test from logistic regression (AUC = 0.606) was slightly better than that from the robust optimal ROC curve method (AUC = 0.596) (Figure 1). If we treat the test obtained from logistic regression as our proposed test, then the proposed test had a slightly higher classification accuracy than the previous three-marker test (AUC = 0.58) [2], though the improvement was not significant (*p*-value = 0.25). We also compared the discriminative ability of our test with that from Lango et al. [3] by using the test statistics introduced in the Methods section and setting *A*_0 _equal to 0.6 (i.e., the discriminative ability obtained by Lango et al.); in the FHS no strong evidence was found to suggest a difference of classification accuracy between the two tests (*p*-value = 0.44).

There is evidence that genetic variants might play important roles in causing early-onset T2D [6]. Similar to a previous study [10], we chose 50 years as the cutoff for early- and late-onset T2D. By using the same logistic regression model, we formed two predictive genetic tests to investigate the impact of these 18 genetic variants separately on early- and late-onset T2D. The first test was built to discriminate between patients with T2D onset at less than 50 years old from the rest of the T2D patients and healthy individuals, and the second test was built to discriminate between patients with T2D onset at greater than or equal to 50 years old from the rest of the T2D patients and healthy individuals. The AUC of the former test was 0.683, which is significantly higher than the AUC of the proposed T2D test; and the AUC of the latter predictive genetic test was 0.616. To estimate the predictive values, we created a dataset by randomly selecting one individual from each family without knowing case status, and then applied the proposed T2D test to all 1231 individuals to assign each individual a risk score. For ease of interpretation, we grouped all individuals into three risk subgroups (i.e., low risk, medium risk, and high risk) based on the tertiles of their risk scores. For each risk subgroup, we estimated the positive predictive values by calculating the proportion of individuals within the subgroup having T2D. For individuals with low risk, medium risk, and high risk genotype profiles, their chance of developing T2D was found to be 7.48%, 7.60%, and 17.13%, respectively. The same strategy was also applied to the test for determining early-onset T2D, and the chance of developing early-onset T2D for the three risk subgroups was 0.38%, 1.96%, and 3.59%, respectively.

## Discussion

Ongoing GWAS provide a powerful approach to uncovering common unknown genetic variants causing complex diseases. The discovered genetic variants, together with previous findings, not only improve our understanding of the genetic etiology of common complex diseases, but also may create new opportunities to improve health care by making early disease prediction possible.

In this study, we combined 18 loci to form a predictive genetic test for T2D from FHS data. Our findings were consistent with previous studies that suggested a limited improvement of the test by incorporating an additional 15 loci into the previous test based on three loci. That this success was limited can be attributed to at least two causes. First, the currently discovered genetic variants only explain a small proportion of the genetic contribution to T2D [11]. Second, as we previously suggested [9], environmental risk factors (e.g., diet) and gene-environmental interactions may play important roles in causing T2D. We were unable to investigate these effects in this FHS dataset because environmental risk factors were not available. In the future, by incorporating additional genetic variants, environmental risk factors, and their interaction effects, we could gradually improve the classification accuracy of the T2D test, and this may eventually lead to a clinically useful test.

We found the 18 loci predisposing to T2D had substantially better ability to detect patients with early-onset T2D than to detect patients with late-onset T2D. This result, although still preliminary, suggests a promising predictive genetic test for early-onset T2D. Our result also confirms a previous hypothesis suggesting there is a major genetic influence on early-onset T2D, but that joint effects of environmental and genetic risk factors influence on late-onset T2D.

In additional to logistic regression, we also applied our newly developed optimal robust ROC curve method in this study. Although, in this specific case, there seemed to be no additional accuracy in using the optimal robust ROC curve method, the new method may potentially increase a test's performance in other scenarios in which there is gene-gene interaction. Thus, depending on the nature of the disease etiology, an appropriate method could be chosen. For a simple scenario involving a relatively small number of loci with no gene-gene interaction expected, logistic regression would be preferred. When there is a large number of loci with high order interactions, the optimal robust ROC curve method should be applied instead.

## Conclusion

Using the FHS dataset, we found little increase in discriminative ability as a result of combining 15 additional T2D loci discovered from the recent GWAS, together with three already known loci, to form a predictive genetic test. Although the currently identified T2D loci do not have sufficient power for predicting T2D, they may have a larger impact on early-onset T2D, and hence could be used to predict early-onset T2D.

## List of abbreviations used

AUC: Area under the ROC curve; FHS: Framingham Heart Study; GWAS: Genome-wide association studies; ROC curve: Receiver operating characteristic curve; SNP: Single-nucleotide polymorphism; T2D: Type 2 diabetes

## Competing interests

The authors declare that they have no competing interests.

## Authors' contributions

QL and RCE conceived the idea and participated in its design and coordination. QL, YS, and XW carried out programming and analyses. SW and YC contributed to the initial conception and design and provided critical revisions of the manuscript. All authors read and approved the final manuscript.

## References

[B1] Larkin M Diet and exercise delay onset of type 2 diabetes, say US experts Lancet 2001 358 565 10.1016/S0140-6736(01)05751-8 11520536

[B2] Weedon MN McCarthy MI Hitman G Walker M Groves CJ Zeggini E Rayner NW Shields B Owen KR Hattersley AT Frayling TM Combining information from common type 2 diabetes risk polymorphisms improves disease prediction PLoS Med 2006 3 e374 17020404 10.1371/journal.pmed.0030374 PMC1584415

[B3] Lango H UK Type 2 Diabetes Genetics Consortium Palmer CN Morris AD Zeggini E Hattersley AT McCarthy MI Frayling TM Weedon MN Assessing the combined impact of 18 common genetic variants of modest effect sizes on type 2 diabetes risk Diabetes 2008 57 3129 3135 18591388 10.2337/db08-0504 PMC2570411

[B4] van Hoek M Dehghan A Witteman JC van Duijn CM Uitterlinden AG Oostra BA Hofman A Sijbrands EJ Janssens AC Predicting type 2 diabetes based on polymorphisms from genome-wide association studies: a population-based study Diabetes 2008 57 3122 3128 18694974 10.2337/db08-0425 PMC2570410

[B5] Lu Q Obuchowski N Won S Zhu X Elston RC Using the optimal robust receiver operating characteristic curve for predictive genetic tests Biometrics in press 10.1111/j.1541-0420.2009.01278.x PMC3039874 19508241

[B6] Hanson RL Elston RC Pettitt DJ Bennett PH Knowler WC Segregation analysis of non-insulin-dependent diabetes mellitus in Pima Indians: evidence for a major-gene effect Am J Hum Genet 1995 57 160 170 7611284 PMC1801224

[B7] Peduzzi P Concato J Kemper E Holford TR Feinstein AR A simulation study of the number of events per variable in logistic regression analysis J Clin Epidemiol 1996 49 1373 1379 10.1016/S0895-4356(96)00236-3 8970487

[B8] Egan JP Signal Detection Theory and ROC Analysis 1975 New York, Academic Press

[B9] Lu Q Elston RC Using the optimal receiver operating characteristic curve to design a predictive genetic test, exemplified with type 2 diabetes Am J Hum Genet 2008 82 641 651 18319073 10.1016/j.ajhg.2007.12.025 PMC2664997

[B10] Aguilar-Salinas CA Reyes-Rodríguez E Ordóñez-Sánchez ML Torres MA Ramírez-Jiménez S Domínguez-López A Martínez-Francois JR Velasco-Pérez ML Alpizar M García-García E Gómez-Pérez F Rull J Tusié-Luna MT Early-onset type 2 diabetes: metabolic and genetic characterization in the Mexican population J Clin Endocrinol Metab 2001 86 220 226 10.1210/jc.86.1.220 11232004

[B11] Zeggini E Weedon MN Lindgren CM Frayling TM Elliott KS Lango H Timpson NJ Perry JR Rayner NW Freathy RM Barrett JC Shields B Morris AP Ellard S Groves CJ Harries LW Marchini JL Owen KR Knight B Cardon LR Walker M Hitman GA Morris AD Doney AS Wellcome Trust Case Control Consortium (WTCCC) McCarthy MI Hattersley AT Replication of genome-wide association signals in UK samples reveals risk loci for type 2 diabetes Science 2007 316 1336 1341 10.1126/science.1142364 17463249 PMC3772310

